# Development and Validation of the High-Voltage Direct-Current Modular Multilevel Converter (HVDC-MMC) Model for Converter Transformer Protection Studies

**DOI:** 10.3390/s24103126

**Published:** 2024-05-14

**Authors:** Krzysztof Solak, Waldemar Rebizant, Frank Mieske

**Affiliations:** 1 Faculty of Electrical Engineering, Wroclaw University of Science and Technology, 50-370 Wrocław, Poland; krzysztof.solak@pwr.edu.pl; 2 Siemens AG, 13599 Berlin, Germany; frank.mieske@siemens.com

**Keywords:** inverter-based resources (IBRs), converter transformer, differential protection, modular multilevel converter (MMC), low-voltage ride-through (LVRT), fast fault injection, transient simulation

## Abstract

The electrical protection of power networks with fault contribution from inverter-based power sources imposes new application challenges that have to be dealt with by protection engineers. This paper describes the development of a study case model of an HVDC-MMC link for testing the protection behaviour of connected converter transformers. The paper summarises the implementation and validation of the converter control as well as enhancements to provide Fault Ride-Through capability and fast fault current injection as required by the German Technical Connection Rules for HVDC. The grid code standard requires positive- and negative-sequence reactive current injection in the case of grid faults. A Doubled Decoupled Synchronous Reference Frame Phase Locked Loop (DDSRF-PLL) for Vector Current Control (VCC) is implemented. Additionally, a Fault Detection and Fault Ride-Through Reference Generator with a Current Limitation strategy is introduced. Though these techniques are well described in the literature, the DDSRF is improved for current control stability. The relationship between the parameters of the PLL and the control, as well as the behaviour of the protection system, are demonstrated. Grid faults with large voltage dips pose a significant challenge to the stability of the control system. Nevertheless, it is shown that with the developed model, it is possible to make general statements about the protection behaviour in an inverter-based environment.

## 1. Introduction

Given the goal to achieve a climate-neutral Europe, wind—especially offshore wind energy—is key to the EU Commission’s renewable energy plan. By 2050, Europe aims to expand offshore wind energy to 300 GW [1,2]. Offshore wind energy is produced in large Wind Power Plants (WPPs), with wind turbines located far away from land. If the distance from offshore to onshore is greater than 100 km, then the High-Voltage Direct-Current (HVDC) transmission link is the only feasible technical solution [3]. For the transformation of the direct current to the alternating current and vice versa, power electronic Voltage Source Converters (VSCs) with power transformers are used in the offshore and onshore substations, as described in [4]. The VSC technology is particularly advantageous for linking with offshore WPPs in comparison to classical Line Commutated Converters (LCCs). The state-of-the-art VSC technology is the Modular Multilevel Converter (MMC) in half-bridge topology [5].

**Figure 1:**
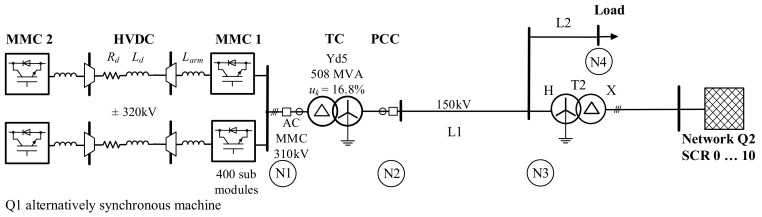
Single line diagram of the study case test power system model with the HVDC-MMC link.

To obtain the transient data for the HVDC-MMC converter transformer protection study, a digital electromagnetic transient model, the Simscape Power Systems [6] MATLAB Simulink model, was developed, corresponding to the single line diagram shown in Figure 1. It comprises the considered HVDC-MMC, the converter transformer TC, an AC transmission line L1, a load, and a second equivalent power source Q2 with transformer T2. The AC transmission system is grounded by the transformer star points. The main objective of the simulation with the test system is to investigate the interaction of the HVDC-MMC current control with external faults and transformer internal faults.

The strength of the receiving-end AC power source Q2 is varied in the simulations. It is characterised by the Short-Circuit Ratio (SCR), defined in [7] as the ratio of the receiving-end power to the rated power of the converter P_rC_ at the nominal voltage at the Point of Common Coupling (PCC):

(1) $$SCR=\left.\frac{S_{Q2}}{P_{rC}}\right.$$

If the receiving-end AC power source Q2 is disconnected, the SCR is 0. Thus, the power system is powered only from the HVDC link and characterised by pure inverter-based generation. To guarantee the stability of the energy network, a regulatory framework of the Transmission Network Operators (TNOs) is defined. The European Network of Transmission System Operators for Electricity (ENTSO-E) has implemented network codes for the Europe-wide harmonisation of the requirements for the TSO networks and the replacement of national standards. The standard [8] defines general requirements for active power control and frequency support, reactive power control and voltage support, Fault Ride-Through (FRT) or Low-Voltage Ride-Through (LVRT) capability, control, protection devices and settings, and power system restoration. The transmission code for the German transmission system is found in [9,10]. The ability of electric generators to stay connected during short-time voltage drops due to AC faults is referred to as the LVRT capability. During AC faults, the generation unit should remain connected and support the grid with reactive power. The targets of AC voltage support with Fast Fault Reactive Current Injection (FFCI) during balanced and unbalanced faults are increasing the positive-sequence voltage, decreasing the negative-sequence voltage, suppressing the zero-sequence voltage, reducing the overvoltage of the healthy phases, and increasing the voltage of the faulty phases at the PCC [11].

The MMC is equipped with Quadrature Current Injection (DQCI) with Phase Locked Loop (PLL)-type controls. There is no physical coupling between positive- and negative-sequence current injection. The detailed FFCI characteristic is summarised in a guidance document [12] of the expert group FFCI in the ENTSO-E. In the following, the requirement of positive-sequence current injection will be referred to as Positive-Sequence Injection Low-Voltage Ride-Through (PSI-LVRT), and the negative-sequence current injection as Negative-Sequence Injection Low-Voltage Ride-Through (NSI-LVRT) [13]. With respect to these requirements, this paper presents the experiences and necessary adaptations of standard MMC control [13,14] and modelling approaches to comply with the fast fault current injection during balanced and unbalanced faults.

Efficient and accurate modelling and simulation of Modular Multilevel Converters (MMCs) are the basis for analysing the behaviour of transformer differential protection in a high-voltage and high-capacity HVDC-MMC system. The simulation model to investigate transformer protection is expected to comply with the following requirements: accurate response to unbalanced external grid conditions, converter transformer faults, blocking behaviour of MMC, short-circuit current provision, reactive Fast Fault Current Injection (FFCI) by LVRT control (reactive positive- and negative-sequence current injection), converter harmonics influencing protection (<10th harmonic), circulating current phenomena, and MMC submodule voltage ripples.

In MATLAB/Simulink, no standardised simulation model for the HVDC-MMC is available that meets the requirements listed above. Therefore, an adequate HVDC-MMC simulation model is necessary. Development of such a model was a huge challenge, since modelling MMC and all its control functions is very complex.

The most accurate modelling would include the models of the Insulated Gate Bipolar Transistors (IGBTs) and the diodes of each submodule physic in detail, with differential equations. The simulation accuracy provided by this is closest to the actual system. The number of submodules on a single HVDC-MMC arm is several hundred, so the simulation process would be very slow. The other detailed models are based on simplified nonlinear IGBT models or simplified switchable resistances. The charge and discharge process of each submodule capacitor is modelled. Such a model is implemented in the MATLAB Simulink Specialised Power System [6] as the switching device type for the half-bridge MMC. It is used in the course of this study only for validation of the model. More efficient modelling of the half-bridge MMC aggregates the submodules of the six converter arms into one equivalent module. A controlled voltage source and a current source are used within the average model. The current source forms the sum of the voltages across the capacities via the arm capacity. So, the simulation speed is not restricted by the number of cascaded submodules. The representatives are the continuous model, the average value model, the nonlinear average model, and the arm-level averaged model (ALA) with blocking capability. With the aggregate model, it is assumed that the capacitor voltages of all power modules are balanced by a subordinate regulation. However, the dynamics of the control system, the harmonic content of the converter, the vertical energy flow, and the horizontal energy flow, with the phenomenon of circular currents, are well represented. Therefore, the continuous average model is used in particular for the dynamic analysis of the relationship between the sum capacitance voltages and the horizontal and vertical energy exchange between the converter arms and their control algorithms. In the developed study case, the HVDC-MMC model and the Simulink Specialised Power System Half-Bridge Aggregate model with Pulse Width Modulation (PWM) control are used.

It was assumed that the MMC controller should contain the following functions: Vector Current Control (VCC), AC voltage control (AVC), and active power control for steady-state operation; Circulating Current Suppression Control (CCSC) and arm energy control; and Doubled Decoupled Synchronous Reference Frame PLL (DDSRF-PLL) and low-voltage ride-through (LVRT). These were all developed with adequate settings in MATLAB/Simulink. The following sections detail the design of the MMC control for grid-compliant modelling for protection studies.

## 2. Theoretical Background

**Figure 2:**
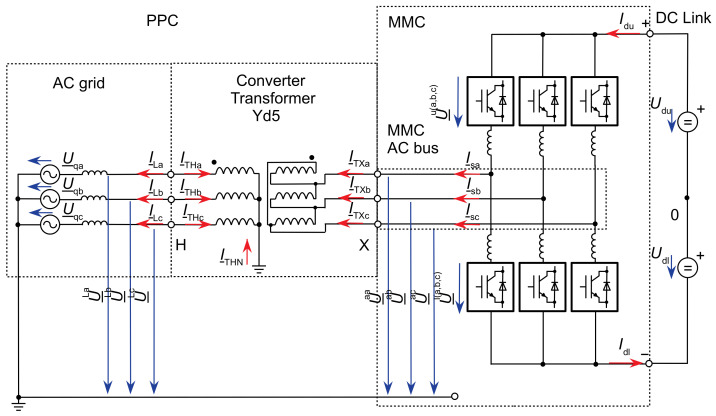
Equivalent circuit of the HVDC-MMC and the converter transformer, voltages, and currents in active sign convention.

The output of the MMC is delivered through the converter transformer to the PCC to the power system. The currents and voltages at the AC bus of the converter and the PCC are defined by the active-sign convention, as shown in Figure 2.

The positive-sequence voltage deviation at PCC is

(2) $$∆U_{1L}=U_{1L}^{F}-U_{1L}^{b}$$

where $U_{1L}^{F}$ is the fault positive-sequence voltage at the PCC (converter grid side) and $U_{1L}^{b}$ is the average voltage before fault.

**Figure 3:**
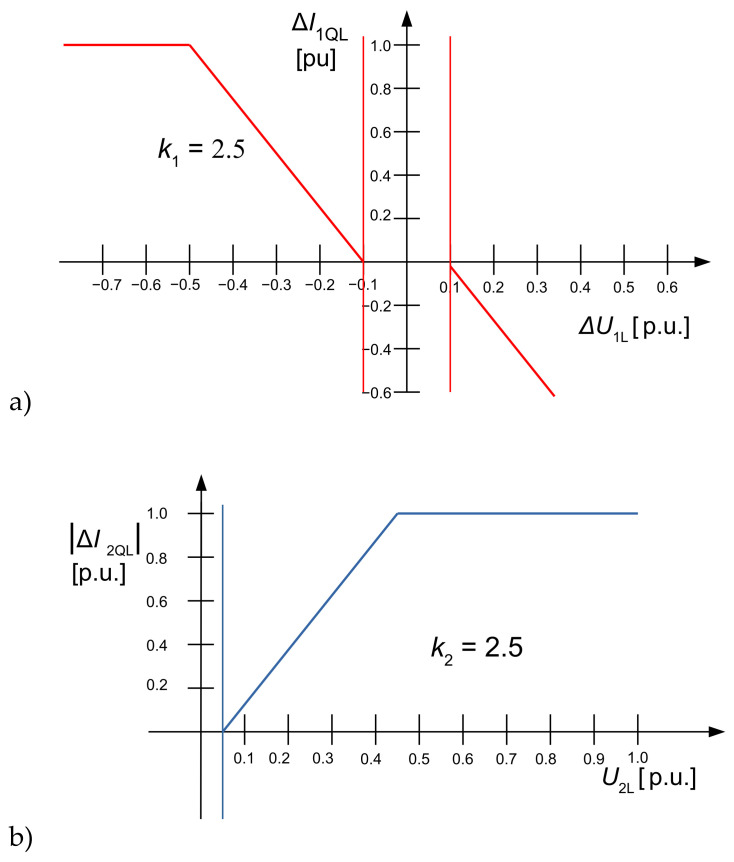
LVRT reactive positive- and negative-sequence current injection characteristics in active sign convention, VDE4120 [10]: (**a**) LVRT reactive positive-sequence current injection characteristic, (**b**) LVRT reactive negative-sequence current injection characteristic.

As shown later, deadbands of minimum voltage deviations to trigger the FRT functionality have a significant impact on the behaviour of transformer protection in the case of low fault currents. The ordinance for wind power plants [15] defines the term relevant voltage deviation $∆U_{1LR}$, $∆U_{2LR}$ for positive- and negative-sequence voltage. If $∆U_{1LR}$, $∆U_{2LR}$ deviates only within a defined deadband, the relevant voltage deviation is set to zero. The required additional reactive current in positive-sequence system $∆I_{1QL}$ shall be proportional to a predefined droop factor k_1_ (3), as shown in Figure 3a. In Germany, the guideline VDE-AR-N 4120 [2] requires an additional reactive negative-sequence current $∆I_{2QL}$ proportional to the negative-sequence voltage at the PCC (as shown in Figure 3b) according to (4).

(3) $$∆I_{1QL}=jk_{1}∆U_{1LR}$$

(4) $$∆I_{2QL}=j\;k_{2}\;U_{2R}$$

**Figure 4:**
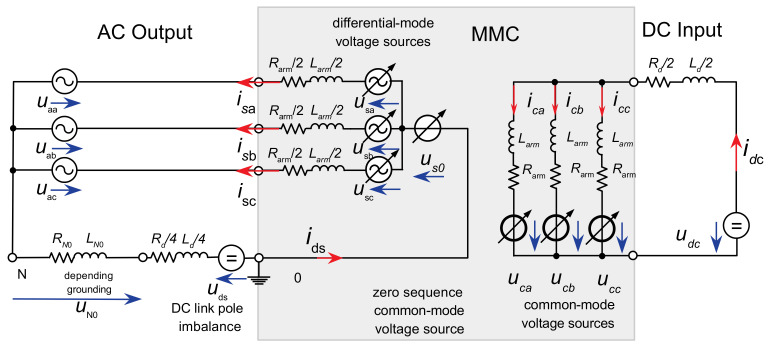
Decoupled equivalent circuits and voltage sources for the AC and DC side of the MMC.

For modelling the dynamics of the MMC control, the average value model ([13,14,16,17,18]) with decoupling of the AC and DC sides of the MMC in controllable voltage sources is applied, as shown in Figure 4.

The voltage source $u_{s}={\left[u_{sa},u_{sb},u_{sc}\right]}^{T}$ in the differential mode is formed from the difference of the inserted arm voltages and constitutes the inner AC emf of the MMC and drives the AC side output current vector behind the converter arm and converter transformer leakage impedance. The converter inner AC emf is set by a Vector Current Control (VCC) inherited by field-oriented control of AC drives [19].

**Figure 5:**
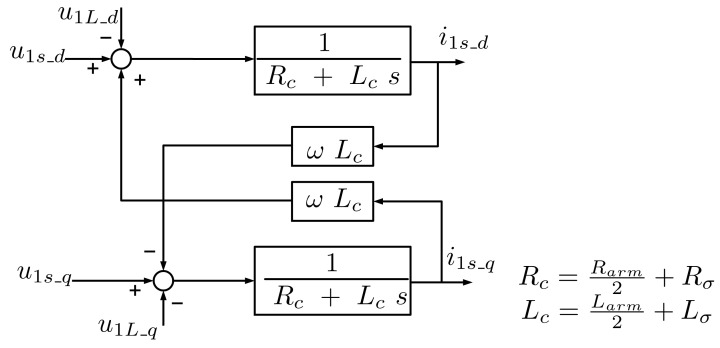
Transfer function block diagram of the AC MMC current control plant in the positive-sequence synchronous dq reference frame.

Space Vector Current Control (dq current control) is a well-known current control technique for three-phase currents that uses a rotating synchronous reference frame synchronised with the grid voltage. The block diagram of the transfer function of the AC output equivalent to the differential equation of the HVDC MMC current control plant in the positive-sequence dq-synchronous reference frame is shown in Figure 5.

Grid synchronisation is the process of transforming the measured instantaneous samples of the three-phase grid voltage into a positive- and negative-sequence component space vector $u_{1L\_dq}$, $u_{1L\_dq}$ in two dq-synchronous reference frames, and vice versa, to the output control values. A Decoupled Double Synchronous Reference Frame PLL (DDSRF-PLL) synchronises the synchronous reference frame to the grid voltage [20]. Especially in the case of unbalanced grid faults and weak grids, the extraction of the sequence components, the harmonic filtering technique, and the obtained corresponding phase margin are essential for the stability of the inverter control.

**Figure 6:**
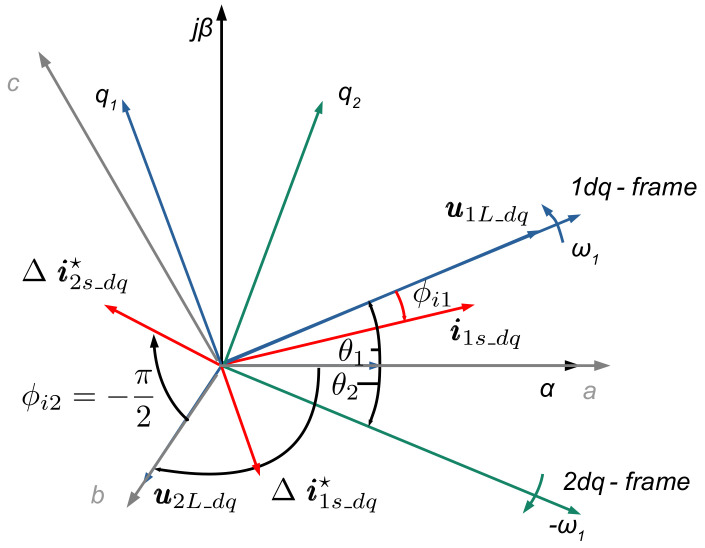
Vector diagram of the positive- and negative-sequence space vectors of the grid voltage u_1L_dq_, u_2L_dq_ and converter control reference value i*_1s_dq_ and reference values for fast fault reactive current injection Δi*_1s_dq_, Δi*_2s_dq_.

Figure 6 shows the positive- and negative-sequence space vectors of grid voltage $u_{1L\_dq}$, $u_{1L\_dq}$ and converter current control reference values $i_{1s\_dq}^{*}$, i*_2s_dq_ in the dq-synchronous reference frames. The grid voltage positive-sequence space vector is aligned with the phase-locked loop (PLL) to the d-axis and, therefore, has a zero-degree phase shift. The positive-sequence grid voltage space vector is rotated by 5/6π, and the negative-sequence grid voltage by −5/6π due to the converter transformer vector group compensation, which is shown in the figure accordingly. In this way, the converter current phase shift angles can be directly related to the grid voltage. Consequently, the positive-sequence instantaneous active and reactive powers are directly related to the positive-sequence dq components.

## 3. Development of HVDC-MMC Control

The MATLAB/Simulink Simscape Power Systems [5] model corresponding to the system shown in Figure 1 was developed to obtain the transient data for investigation of the converter transformer differential protection. It includes the HVDC link, the offshore MMC with a rated transmission capacity of 690 MVA, and a DC pole-to-pole voltage of 640 kV. The transformer is a 508 MVA, 310/166 kV three-phase transformer with converter winding connected in delta to block the zero-sequence voltages generated by the MMC.

**Figure 7:**
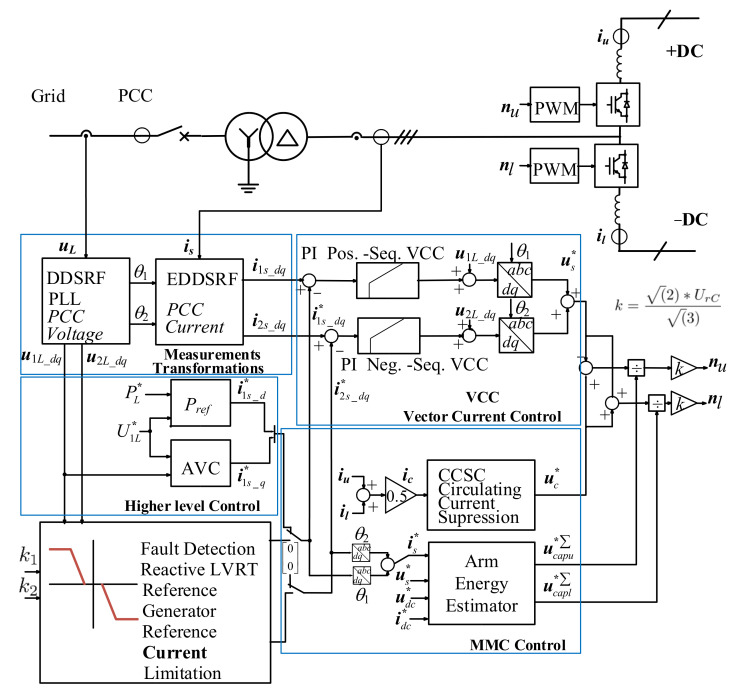
HVDC-MMC control layout for the test power system.

The MMC is designed with Insulated-Gate Bipolar Transistor (IGBT) devices, 400 submodules (SM) per arm, and an arm inductance of 50 mH or 0.33 p.u. A submodule capacitance of 10 mF was chosen based on the proposal of the energy storage requirement of the MMC total energy of 40 kJ/MW, as described in [21,22]. The used half-bridge MMC model was an arm-level averaged (ALA) model with blocking capability [23]. With the aggregate model, it is assumed that the capacitor voltages of all power modules are balanced by a subordinate regulation. However, the dynamics of the control system, the harmonic content of the converter, the vertical energy flow, and the horizontal energy flow with the phenomenon of circular currents are well represented. The HVDC-MMC control layout and the measured, reference, and output values for the inverter-based generation test power system are outlined in Figure 7. The control hierarchy of the MMC consists of the higher level control, the Vector Current Control (VCC), and the MMC internal control. The outer loop sets the reference values for the VCC. The high-level control consists of an AC voltage controller (AVC) and active power control for steady-state operation. To comply with the LVRT requirements, a parallel block generates the positive- and negative-sequence current reference values for the VCC in the event of a fault. The MMC internal control comprises the Circulating Current Suppression Control (CCSC) and the arm energy control. In steady state, the control is designed for the grid-following mode.

**Figure 8:**
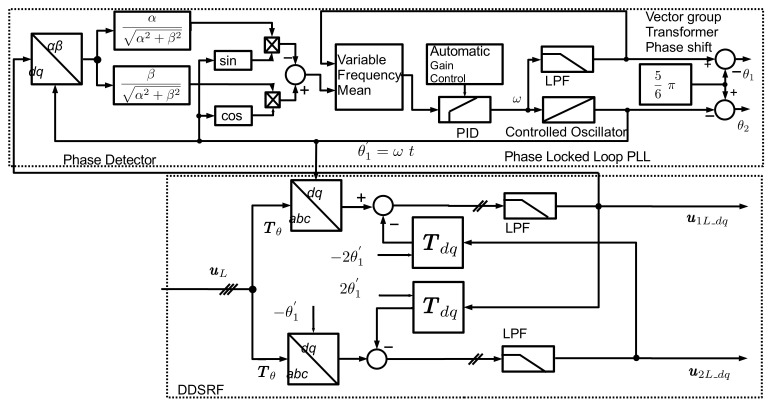
Vector structure of the DDSRF-PLL.

The rotating αβ-space vectors can be brought to a standstill on a Double Synchronous Reference Frame (DSRF) by using the Park transformation. The coupling between the axes and the rotation of the voltage vectors in opposite directions generates undesired second harmonic components from the other sequence component. To eliminate these harmonics without importing a significant group delay of a harmonic filter, the Decoupled Double Synchronous Reference PLL (DDSRF-PLL) presented in [20,24] is used for the grid synchronisation PLL. Figure 8 presents the detailed structure of the PLL with the DDSRF for obtaining the grid voltage dq-space vector. The Simscape PLL, with phase detector, PID controller with Automatic Gain control, and controlled oscillator was embedded in a quadrature signal generator (QSG) based on the q-axis. The low-pass filter after the cross-coupled second harmonic elimination is the first-order filter with a cut-off frequency of $\omega_{f}=\omega_{N}/\sqrt{2}$ according to [25], with a reasonable trade-off between settling time and the second harmonic attenuation.

**Figure 9:**
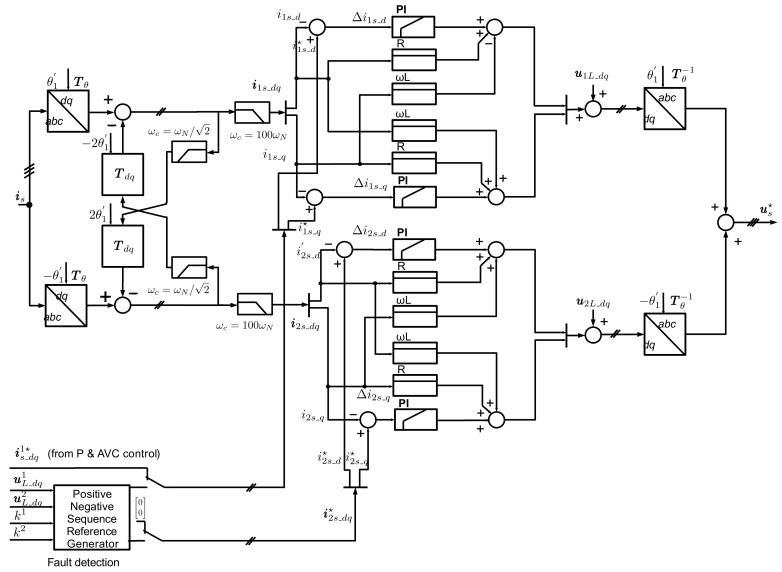
Block diagram of current control in the Enhanced DDSRF (EDDSRF) with two different low-pass filters.

The analyses reveal that the low-pass filter in the feedback loop of the current control loop leads to instability of the current control due to a reduced phase margin. Therefore, an enhanced DDSRF (EDDSRF) was proposed in [24] based not on the measured current but on the current control references. However, stable results could not be obtained with this method since the current reference deviates considerably from the actual values in the case of unsymmetrical faults, especially in a weak grid. An arrangement with a low-pass filter with a low cut-off frequency before the decoupling circuit and a low-pass filter with a high cut-off frequency in the direct feedback loop has proven to be advantageous—see Figure 9. The structure of the EDDSRF and bode-plot of the closed-loop transfer function of VCC is given below. The transfer function of the open-loop system is $H_{cc_{O}L}\left(s\right)=G_{d}\left(s\right)G_{PI}\left(s\right)G_{P}\left(s\right)$, whereas $G_{d}\left(s\right)$ denotes the system computation dead-time, $G_{PI}\left(s\right)$—the PI controller, and $G_{P}\left(s\right)$—the plant transfer function. The cross-coupling effect between the d- and q-axis of the plant transfer function, shown in Figure 5, is eliminated by the proposed control structure and is therefore omitted in the open-loop transfer function. The dead time of the power converter, including pulse-width modulation delay time, processing time, and sampling time, is simplified by a first-order system element (PT1) with time constant τ = 1.5 $T_{S}=1.5\;1/f_{s}$, where f_s_ is the sampling frequency of the controller as described in [26]. The open-loop transfer function is then given by following equation:

(5) $$H_{CC_{O}L}(s)=\frac{k_{ip}\left(1+T_{i}\;s\right)}{T_{i}\;s}\frac{1}{1+{1.5T}_{s}\;s}\frac{k_{c}}{1+T_{c}\;s}$$

where k_ip_, T_i_ are the proportional gain and integral time of the PI controllers, $k_{c}=1/R_{C}$, $T_{C}=R_{C}/L_{C}$ are the gain and time constant of the converter arm reactor and the transformer leakage impedance. The time constant T_c_ of the MMC arm reactor and transformer impedance is around 218 ms and, therefore, significantly greater than the sampling time constant 1.5 T_s_ of around 0.5 to 1 ms. Thus, the modulus optimum method is applied with the choice of the PI integrator time constant T_i_ equal to the plant time constant T_c_, and thus, the transfer function for the open-loop system is obtained as

(6) $$H_{CC_{O}L}(s)=\frac{{2k_{c}\;k}_{ip}}{{3T}_{c}T_{s}s^{2}+T_{c}\;s}$$

Based on the open-loop transfer function, the phase margin was calculated at 78.3°, while the gain margin is infinite, which means that the closed-loop system is stable.

Without taking into account the cut-off frequency of the DDSRF low-pass filter, one obtains the second-order transfer function for the control loop:

(7) $$H_{CC_{C}L}\left(s\right)=\frac{H_{CC_{O}L}\left(s\right)}{{1+H}_{CC_{O}L}\left(s\right)}=\frac{{2k_{c}\;k}_{ip}}{{3T}_{c}T_{s}s^{2}+2T_{c}\;s+{2k_{c}\;k}_{ip}}$$

For an optimal damped system with 5 per cent overshoot in response to a reference step change, the proportional gain of PI control is $k_{ip}=T_{c}/\left(2k_{c}\;T_{s}\right)$. For completeness, the closed-loop transfer function with the DDSRF low-pass filter in the feedback loop is given as

(8) $$H_{CC_{C}Lf}(s)=\frac{H_{CC_{O}L}\left(s\right)}{{1+H}_{CC_{O}L}\left(s\right)\frac{1}{1+T_{f}\;s}}$$

**Figure 10:**
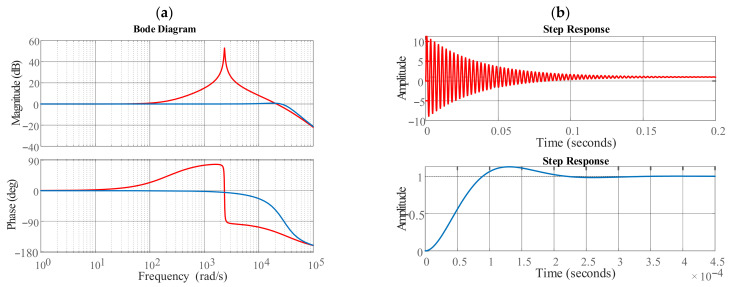
Bode plot (**a**) and step response (**b**) of the closed-loop current control transfer function.

Figure 10a shows the bode plot of the closed-loop current control transfer function with a DDSRF low-pass filter of $T_{f}=\sqrt{2}/\omega_{N}$ (red line) and the implemented low-pass filter of $T_{f}=1/{(100\;\omega }_{N})$ (blue line). For the first DDSRF low-pass filter, the magnitude characteristic shows a sharp rise with a simultaneous drop in the phase of 180 degrees at 2350 rad/s. In contrast, for the second DDSRF low-pass filter, this rise is in the range of the first-order time delay cut-off frequency and therefore it has negligible influence on the dynamics of the current control loop. Figure 10b presents step responses for the closed-loop current control transfer function with a DDSRF low-pass filter for two values of T_f_. Very low settling time and small overshoot is observed for the second DDSRF low-pass filter (see Figure 10b, blue line)—this result proves that PI controller settings are correctly matched and the proposed low-pass filter with high cut-off frequency was properly designed.

It can be concluded that the MMC control depends on correctly determining the space vectors of grid voltage and current in the synchronous reference frame. The correct determination of grid voltage and current positive and negative space vectors is ensured by the Decoupled Double Synchronous Reference Frame (DDSRF-PLL). However, the original low-pass filter in the DDSRF degraded the control characteristics of the current control, so the DDRSF had to be optimised. The above analysis shows that the proposed method with a low-pass filter with a high cut-off frequency gives satisfactory results. However, there is still room for improvement/optimisation of the performance of this algorithm; further research will be conducted in this direction in the future.

### 3.1. Implementation of LVRT Control

The LVRT control is implemented based on the grid code requirements for reactive positive- and negative-sequence current contributions described in the theoretical background and implementation descriptions in [13,19,27]. The LVRT control logic comprises fault detection and drop-out logic, as well as positive- and negative-sequence current reference generation and current reference limiting. The fast fault current provision time impacts protection operating time and should, according to VDE-AR-N 4120 [10], not exceed a maximum step response time of 30 milliseconds.

The positive-sequence voltage deviation is determined as the difference between the actual and the pre-fault positive-sequence voltage according to (2) implemented with the absolute value of the dq vector:

(9) $$\begin{array}{c} \Delta \;U_{1L}\left(k\right)=\left|u_{1L\_dq}\left(k\right)\right|\;\;\;-\;\;\;\mathrm{MEAN}\left|u_{1L\_dq}\left(k-M\right)\right| \end{array}$$

(10) $$\begin{array}{c} ∆\;i_{1s\_q\_req}^{*}=∆\;i_{1s\_q}^{*}\;\;-∆\;i_{1s\_q\_req\_AVC\_frozen}^{*} \end{array}$$

This summation is essential because, in the event of a fault, voltage deviation of slightly above 0.1 would result in less reactive current being injected than in the operating state of AVC. In addition, the pre-fault state in the AVC control must also be maintained for the post-fault state. An individual negative-sequence voltage deadband of 0.05 p.u. is recommended to ensure correct negative-sequence angle detection. The negative-sequence voltage deviation $\Delta \;U_{2L}$ is obtained similar to (9), and the absolute value of requested injected reactive negative-sequence current is obtained:

(11) $$\begin{array}{c} ∆\;i_{2s\_dq\_req}^{*}=k_{1}\left(\left|\Delta \;U_{2L}\right|-0.05\right)\;\;\;\mathrm{SIGN}\left\{\Delta \;U_{2L}\right\} \end{array}$$

The angle of the requested injected reactive negative-sequence current is then determined according to Figure 6, as follows:

(12) $$\begin{array}{c} ∠∆\;i_{2s\_dq\_req}^{*}=-∠∆\;u_{2L\_dq}^{*}-\frac{\pi }{2} \end{array}$$

A challenge in implementation was also the setting of hysteresis and the dropout logic in the event of fault clearance, as the voltage may rise similarly after the reactive current is injected or after the fault has been cleared. The requested positive-sequence reactive current reference value, the active current reference value, and the requested reactive negative-sequence current are then processed by the reference current limitation algorithm described below.

### 3.2. Current-Limiting Strategy of LVRT Control

In order not to overload the IGBTs of the MMC, the phase currents on the converter arms must be limited to the effective current I_max_ of 1.2 p.u. Consequently, if a phase current exceeds the above limit, the active current must be reduced first, and if this is not sufficient, the reactive current must also be reduced. The concept of phase current limitation in the LVRT control logic was adopted from [14,28]. The magnitudes of $i_{s}={\left[i_{sa},i_{sb},i_{sc}\right]}^{T}$ converter currents can be calculated from the requested sequence components current reference values ${i_{1s\_dq}^{*},i}_{2s\_dq}^{*}$ by the LVRT reference generator using the inverse Clarke and Park transformation applied on the positive- and negative-sequence components in the dq-synchronous reference frame, according to

(13) $$\begin{array}{c} \begin{array}{c} I_{sa}=\sqrt{\left|i_{1s\_dq\_req}^{*}\right|+\left|i_{2s\_dq\_req}^{*}\right|+\left|i_{1s\_dq\_req}^{*}\right|\left|i_{2s\_dq\_req}^{*}\right|\cos\alpha }\\ I_{sb}=\sqrt{\left|i_{1s\_dq\_req}^{*}\right|+\left|i_{2s\_dq\_req}^{*}\right|+\left|i_{1s\_dq\_req}^{*}\right|\left|i_{2s\_dq\_req}^{*}\right|\cos\left(\alpha -\frac{4}{3}\pi \right)}\\ I_{sc}=\sqrt{\left|i_{1s\_dq\_req}^{*}\right|+\left|i_{2s\_dq\_req}^{*}\right|+\left|i_{1s\_dq\_req}^{*}\right|\left|i_{2s\_dq\_req}^{*}\right|\cos\left(\alpha +\frac{4}{3}\pi \right)} \end{array} \end{array}$$

where $\alpha =∠i_{1s\_dq\_req}^{*}+∠i_{2s\_dq\_req}^{*}$.

The procedure for the determination of required setpoints is as follows:$i_{1s\_d}^{*}=\left\{\begin{array}{c} i_{1s\_d\_req}^{*}\\ {F_{d\_lim}i}_{1s\_d\_req}^{*}\\ 0 \end{array}\right.$$i_{1s\_q}^{*}=\left\{\begin{array}{c} i_{1s\_q\_req}^{*}\\ i_{1s\_q\_req}^{*}\\ F_{q\_lim}i_{1s\_q\_req}^{*} \end{array}\right.$$i_{2s\_dq}^{*}=\left\{\begin{array}{c} i_{2s\_dq\_req}^{*}\\ i_{2s\_dq\_req}^{*}\\ F_{q\_lim}i_{2s\_dq\_req}^{*} \end{array}\right.$Case 1 Case 2 Case 3c(14)

The active current reference is the d-component and is included in the positive-sequence setpoint $i_{1s\_dq}^{*}$. If none of the determined phase currents I_sa_; I_sb_; I_sc_ exceeds the value I_max_, the setpoints will not be reduced (Case 1, (14)). In the other case of exceeding, no direct analytical solution of the limit values for the individual reference values is possible.

Therefore, reducing the limit values is carried out iteratively. Then, (13) is used only with the requested reactive current values to check if the reactive current injection alone exceeds the limit I_max_. If this is the case, the active current reference value is set to zero, and a scaling factor $F_{q\_lim}$ is used to reduce the reactive positive- and negative-sequence current reference values equally (Case 3 in (14)). The scaling factor is obtained by the ratio of I_max_ and the maximum phase current with pure reactive current injection. The available active current reference value or the scaling factor $F_{d\_lim}$ is determined iteratively if the solely reactive current injection does not exceed the limit value. The first starting point is the reduction factor for the active current reference with the difference between *I*_max_ and the obtained maximal current with solely reactive current injection. Then, the maximum current is calculated repeatedly with (13), and the active current is gradually reduced until the maximum current is not exceeded. In (14), this is represented as Case 2.

## 4. Validation of the LVRT Control

Verifying the test system model ensures the validity of the study of transformer protection in the context of inverter-based generation. One should be aware that the detailed behaviour of the MMC LVRT control in the event of disturbances depends very much on the specific, manufacturer-dependent software algorithms. The behaviour can vary significantly according to the software version in the system. The algorithms are constantly improved from experience gained during malfunctions, and new software updates are installed. Therefore, the model verification based on a real disturbance is not targeted. Validation is based on dynamic performance and compliance with the grid code and LVRT requirements summarized in Section 2 and publications. Before the overall simulation model was validated, the network elements were verified individually. The transformer models were validated with short-circuit, no-load, and inrush tests. The verification of the model of the test system includes verification in the operating mode and verification of the LVRT reactive fast fault current injection.

### 4.1. Verification of the HVDC-MMC Simulation Model in Operational Mode

If the MMC is completely de-energized, a start-up procedure is required to charge the MMC submodule capacitors and the DC link capacitance. This is completed using a two-stage start-up procedure. Firstly, the circuit breaker connects the converter to the grid. The antiparallel diodes in the MMC rectify the current. Without charging resistors, a large inrush current would flow into the MMC-HVDC. In the second stage, when the MMC controller is unlocked, the pulse width modulation delivers gate pulses to the submodules, and the DC link voltage finally reaches the nominal value. In the Wind Power Plant application, after the MMC-HVDC line voltage is stabilised, the offshore MMC is unblocked. Then, the offshore MMC control ramps up the reference voltage, and the offshore voltage gradually builds up.

**Figure 11:**
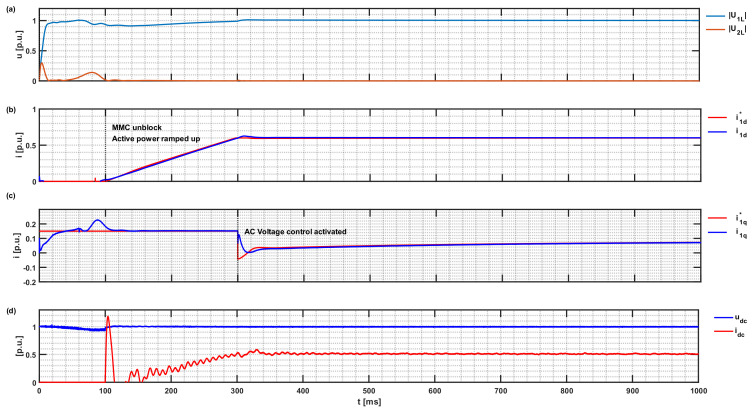
MMC-HVDC active power ramp-up to the operation point: (**a**) PCC voltage: |U_1L_|; |U_2L_|, set point, and actual dq components of injected active and reactive currents in DDSRF, (**b**) i*_1d_; i_1d_, (**c**) i*_1q_; i_1q_, and (**d**) DC-link voltage and current u_dc_; i_dc_.

The simulation starts with the MMC submodules and DC link already energised. Then, after 100 milliseconds, as seen in Figure 11, the MMC controller is unlocked, and the active power is ramped up to the operating point. The voltage regulation to maintain the nominal voltage at the PCC determines injected reactive power. Support for voltage regulation via the on-load tap-changer on the converter transformer is not implemented.

**Figure 12:**
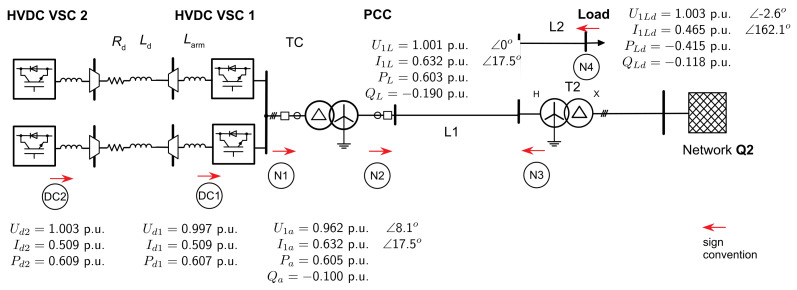
Load flow HVDC-MMC test power system steady state for SCR = 1.

Figure 12 provides the steady-state currents, voltages, and powers at the individual nodes of the study case test system. The voltage angle at the PCC is set to zero. Due to the vector group Yd5 for the two transformers, all voltage and current phasors on the converter side are shown as vector-group compensated to the 155 kV AC network voltage level of the transformers. Examining the power balances between the DC and AC sides of the HVDC-MMC showed a power converter loss of 0.5 per cent. The result agrees with the switching loss and conductor loss evaluation of MMC in [29]. The verification of the total active power losses throughout the interconnected system is thus justified. The power factor on the MMC side is 0.9866 for SCR 1 and 0.954 on the PCC side. The reactive power drop at the grid components agrees with the inductances of the model.

### 4.2. Verification of LVRT Reactive Fast Fault Current Injection

The LVRT reactive fast fault current injection of the HVDC-MMC test model is validated by simulating a phase-to-phase fault and examining the positive- and negative-sequence current space vector dq component step response, instantaneous currents and voltages, and injected current phase displacement angle. A 4 Ω resistive phase-to-phase fault was incepted at grid node N3 (see Figure 1) and cleared after 100 ms through an auxiliary circuit breaker at zero-crossing points of the breaker currents. To demonstrate the correct operation of the internal MMC control in the case of an unbalanced fault and LVRT reactive current injection, the transients of the DC voltage and current, the phase circulating currents, the sum-capacitor voltages of the converter arms, and the horizontal and vertical phase energy were evaluated.

**Figure 13:**
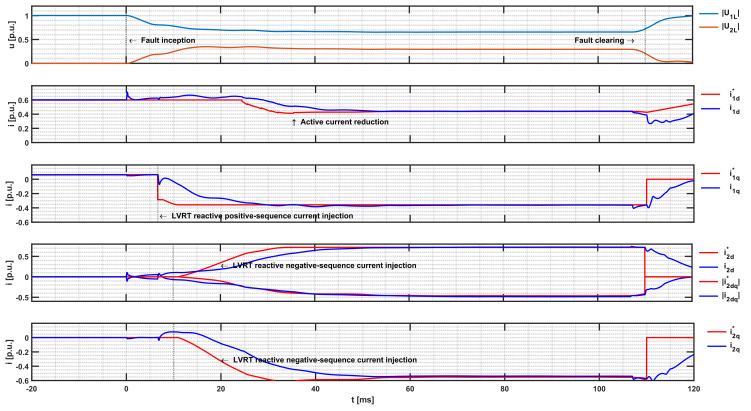
Simulation transient of LVRT fast fault current injection for a phase-to-phase fault at Node 3, SCR = 10.

Figure 13 shows the positive- and negative-sequence voltage at the PCC, the reference and actual values of positive- and negative-sequence current in dq components of the DDSRF in the MMC LVRT, and current control. It can be seen that before the fault inception, the PCC voltage is 1 p.u., the active current component i_1s_d_ is about 0.6 p.u., and the reactive current i_1s_q_ of 0.06 p.u. is set by the active power control and the AVC. The positive-sequence voltage deviation at 7 ms after fault inception is 0.26 p.u. Therefore, after deadband subtraction, the relevant positive-sequence voltage deviation $∆U_{1LR}$ of 0.16 p.u. is given to the LVRT reference generator, and then the requested reactive current reference value $i_{1s\_q}^{*}$ is set to −0.35 p.u. The measured deviation of the negative-sequence voltage at 7 ms after fault inception is 0.2 p.u., and it increases to the final value of 0.34 at 20 ms due to the averaging over a quarter of the power system frequency cycle. By applying (12), the final requested reactive negative-sequence current reference value $i_{2s\_dq}^{*}$ of 0.72 p.u. is obtained at 30 ms after fault inception. The negative-sequence current *q*-component does not correspond to the reference value until another 10 ms later, which is due to the phase shift delay of the feedforward negative-sequence voltage at the negative-sequence *q*-component PI controller, caused by the DDSRF low-pass filter.

**Figure 14:**
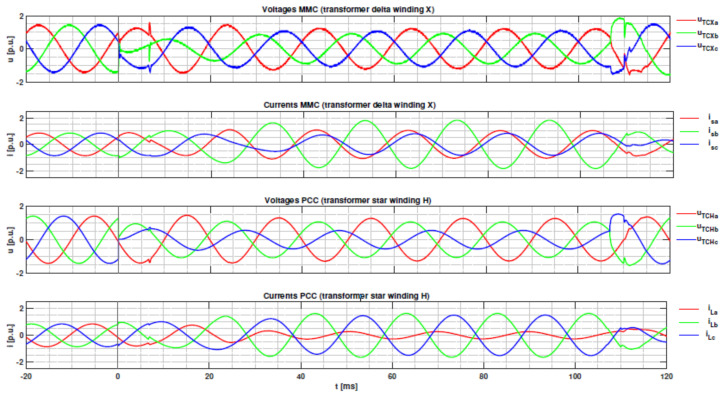
Converter transformer voltages and currents for a simulated for phase-to-phase fault at node N3, SCR = 10.

Without the current limiting strategy, the requested reactive negative-sequence injection reference value would cause an unbalanced AC output current in the converter exceeding the maximum 1.2 p.u. in phase B. Since reactive injection takes precedence, the current limiting strategy will reduce the active current by 0.2 p.u. at 23 ms after fault inception (Case 2 in (14)). It can be seen in Figure 14 that the peak value of the instantaneous AC output current of the inverter in phase B does not exceed 1.7 p.u. and therefore does not exceed the 1.2 p.u. rated current effective value. After the fault clearing, the active current is reset to the pre-fault value using a ramp function.

After fault clearing, the active current is reset to the pre-fault level using a ramp function. The reduction of the active AC output current leads to a corresponding decrease in the DC link current.

**Figure 15:**
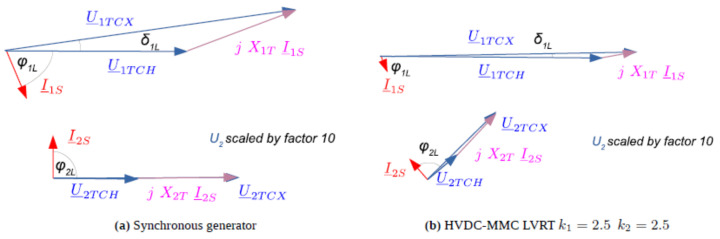
Phasor diagrams of a 4 Ω phase-to-phase fault at node N3 with Q1 synchronous generator (**a**) or HVDC-MMC (**b**) supply with reactive positive-sequence current and additional reactive negative-sequence current injection (40 ms after fault inception).

Table 1 and Figure 15 present the obtained phasor quantities in the case of LVRT fast fault current injection 40 ms after fault inception. To demonstrate the effectiveness of the AC voltage support, test cases with no additional reactive current injection, with reactive positive-sequence injection only, and with reactive positive- and negative-sequence injection were simulated. The results are also reported for a simulation of the same fault with supply in Q1 by a synchronous machine. As can be observed, the positive-sequence PCC voltage U_1TCH_ is the highest with reactive positive-sequence current injection only. The current limiting during positive- and negative-sequence current injection results in a lower positive-sequence PCC voltage than without LVRT control. In this case, however, the negative-sequence voltage is reduced during the fault.

The phase angle displacements of positive-sequence voltage to the negative-sequence current of −83 degrees and negative-sequence voltage to the negative-sequence current of 88 degrees during reactive positive- and negative-sequence current injection are observed in the phasor diagrams in Figure 15b. The phase angle displacements obtained in both configurations correspond as observed in the phasor diagram for a synchronous machine in Figure 15a. This demonstrates that the LVRT fast fault current injection emulates the behaviour of a synchronous machine in the case of unbalanced faults in the converter control. Since the maximum converter current is limited to 1.2 rated current, the positive-sequence current, in particular, is lower than with the synchronous machine.

## 5. Conclusions

It is important to acknowledge that the comprehensive simulation model for the HVDC-MMC system involves numerous parameters and intricate details, making it challenging to precisely mirror the transient response of the actual system. Nevertheless, the simulation model proves valuable in analysing general aspects of the impact of inverter-based generation on protection, as evident from the fault-characteristic transients derived from the implemented HVDC-MMC simulation.

The control algorithms and hardware control of the HVDC-MMC are subject to further development, with the increased use also necessitating algorithm modifications. An IEEE Explore query related to MMC only returned 6450 publications, 1288 of which were published in 2022. Notably, the accuracy of MMC control hinges on the correct determination of the space vectors of grid voltage and current in the synchronous reference frame. Addressing issues such as degraded control characteristics due to original low-pass filters in the DDSRF underscores the need for optimization.

Instances of unbalanced AC faults with high voltage dips in a weak power grid (SCR < 1) led to instabilities in current control. The critical factor here is the impact of AC grid voltage feedforward at the output of the current controller, leading to resonances and oscillations. Disturbances in the correct estimation of the phase angle within the DDSRF-PLL further complicate matters. However, the investigation of turn-to-turn transformer faults with low voltage dips in a weak power system produced plausible results.

Implementing an open-loop sum-capacitor voltage control in the simulation model proved effective for controlling the MMC’s arm energy. It is worth noting that real HVDC-MMC systems incorporate unpublished and more intricate algorithms. In strong AC power grids, the implemented arm energy balancing, coupled with open-loop control of the sum-capacitor voltage, gives satisfactory results in the case of unbalanced faults.

A noteworthy observation pertains to software-specific behaviour, where factors such as the timepoint of the phase jump in fault current during reactive current injection by inverter control depend on the employed measurement filter, fault detection algorithm, and set-point generation algorithm. Despite these complexities, the simulation transients for unbalanced faults align well with those reported in the existing literature.

In a recent technical report by ENTSO-E, titled “High Penetration of Power Electronic Interfaced Power Sources” (HPoPEIPS), the conceptual design of grid-forming converters (GFMs) is outlined [30]. This design incorporates the virtual synchronous machine control (VSM) concept [31,32] for control purposes. Further refinement and development of this model, integrating these advanced control strategies, are essential for conducting future studies of protection behaviour in inverter-based power systems.

## Figures and Tables

**Table 1:** Phasor quantities for a 4 Ω phase-to-phase fault at node N3 with SM and HVDC-MMC supply for various options of LVRT control applied.

	SM	HVDC-MMC		
		w/o LVRT k_1_ = 0, k_2_ = 0	with pos-seq. LVRT k_1_ = 2.5, k_2_ = 0	with pos- and neg-seq. LVRT k_1_ = 2.5, k_2_ = 2.5
U_1TCX_	0.79 p.u. ∠ 7.6°	0.72 p.u. ∠ 10.9°	0.82 p.u. ∠ 9.5°	0.71 p.u. ∠ 1.2°
U_2TCX_	0.23 p.u. ∠ 0°	0.34 p.u. ∠ 20.2°	0.36 p.u. ∠ 20.0°	0.11 p.u. ∠ 44.5°
U_1TCH_	0.49 p.u. ∠ 0°	0.70 p.u. ∠ 0°	0.73 p.u. ∠ 0°	0.62 p.u. ∠ 0°
U2_TCH_	0.49 p.u. ∠ 0.1°	0.35 p.u. ∠ 24.7°	0.36 p.u. ∠ 24.7°	0.27 p.u. ∠ 40.6°
I_1S_	1.40 p.u. ∠ −69.1°	0.61 p.u. ∠ −5.8°	0.72 p.u. ∠ −31.8°	0.46 p.u. ∠ −82.8°
I_2S_	1.18 p.u. ∠ 91.4°	0.13 p.u. ∠ −166.3°	0.14 p.u. ∠ −166.5°	0.77 p.u. ∠ 129.2°

## Data Availability

Data are contained within the article.
